# Genome sequence of the phylogenetically isolated spirochete *Leptonema illini* type strain (3055^T^)

**DOI:** 10.4056/sigs.3637201

**Published:** 2013-05-25

**Authors:** Marcel Huntemann, Erko Stackebrandt, Brittany Held, Matt Nolan, Susan Lucas, Nancy Hammon, Shweta Deshpande, Jan-Fang Cheng, Roxanne Tapia, Lynne A. Goodwin, Sam Pitluck, Konstantinos Liolios, Ioanna Pagani, Natalia Ivanova, Konstantinos Mavromatis, Natalia Mikhailova, Amrita Pati, Amy Chen, Krishna Palaniappan, Miriam Land, Manfred Rohde, Sabine Gronow, Markus Göker, John C. Detter, James Bristow, Jonathan A. Eisen, Victor Markowitz, Tanja Woyke, Philip Hugenholtz, Nikos C. Kyrpides, Hans-Peter Klenk, Alla Lapidus

**Affiliations:** 1 DOE Joint Genome Institute, Walnut Creek, California, USA; 2 Leibniz-Institute DSMZ - German Collection of Microorganisms and Cell Cultures, Braunschweig, Germany; 3 Los Alamos National Laboratory, Bioscience Division, Los Alamos, New Mexico, USA; 4 Biological Data Management and Technology Center, Lawrence Berkeley National Laboratory, Berkeley, California, USA; 5 Oak Ridge National Laboratory, Oak Ridge, Tennessee, USA; 6 HZI – Helmholtz Centre for Infection Research, Braunschweig, Germany; 7 University of California Davis Genome Center, Davis, California, USA; 8 Australian Centre for Ecogenomics, School of Chemistry and Molecular Biosciences, The University of Queensland, Brisbane, Australia

**Keywords:** Gram-negative, flexible, motile, cytoplasmatic tubules, non-sporulating, axial flagella, aerobic, chemoorganotrophic, *Leptospiraceae*, GEBA

## Abstract

*Leptonema illini* Hovind-Hougen 1979 is the type species of the genus *Leptonema*, family *Leptospiraceae,* phylum *Spirochaetes*. Organisms of this family have a Gram-negative-like cell envelope consisting of a cytoplasmic membrane and an outer membrane. The peptidoglycan layer is associated with the cytoplasmic rather than the outer membrane. The two flagella of members of *Leptospiraceae* extend from the cytoplasmic membrane at the ends of the bacteria into the periplasmic space and are necessary for their motility. Here we describe the features of the *L. illini* type strain, together with the complete genome sequence, and annotation. This is the first genome sequence (finished at the level of Improved High Quality Draft) to be reported from of a member of the genus *Leptonema* and a representative of the third genus of the family *Leptospiraceae* for which complete or draft genome sequences are now available. The three scaffolds of the 4,522,760 bp draft genome sequence reported here, and its 4,230 protein-coding and 47 RNA genes are part of the ***G****enomic* ***E****ncyclopedia of* ***Bacteria**** and* ***Archaea***** project.

## Introduction

Strain 3055^T^ was isolated from urine of a clinically healthy bull [1], and was first mentioned in the literature as a new *Leptospira* serotype, serovar *illini* [2,3], but as no name was proposed, it was not validly published. This occurred in the comparative study of Hovind-Hougen [4] who found morphological differences between ‘*Leptospira illini*’ strain 3005 and other members of *Leptospira*, i.e. the presence of cytoplasmatic tubules and the structure of the basal complex of the flagella. These differences, together with the finding of a higher DNA base composition and growth behavior [5] were used as criteria to taxonomically separate strain 3055 from *Leptospira* as *Leptonema illini* with strain 3055^T^ (= DSM 21528 = NCTC 11301) as the type strain. This species is the only species of the genus. The family *Leptospiraceae* was created in the same publication [4], although the name was proposed before, though not effectively published [J Pilot, Ph D Thesis, University of Paris, Paris, France 1965]. Despite a description in the International Journal of Systematic Bacteriology the name *Leptonema* was not included in the Approved List of Bacterial Names [6]. The omission of this name was not in accordance with the *Bacteriological Code* (1990 Revision) Rule 24a, Note 1, but was corrected in Validation List N^o^ 10 [7].

The phylogenetic relatedness among spirochetes and the isolated position of *L. illini* was first elucidated by 16S rRNA cataloguing [8] and then by comparative sequence analysis of reverse-transcribed 16S rRNA sequences [9] and by rDNA analyses [10,11]. The moderate similarity values between *L. illini* and strains of *Leptospira* were later supported by the absence of significant DNA-DNA hybridization values between members of the two genera [12-14], 16S rRNA restriction fragment analysis [15] and PCR amplification of the 16S-23S ribosomal DNA spacer [16]. Application of a 16S rRNA gene real-time PCR assay to leptospiras [17] confirmed the presence of *L. illini* strains in kidneys of Indian rats and bandicoots. Here we present a summary classification and a set of features for *L. illini* strain 3055^T^ together with the description of the complete genomic sequencing and annotation. The rationale for sequencing the genome of this non-pathogenic strain is based on its isolated position within the phylum *Spirochaetes*.

## Classification and features

### 16S rRNA gene sequence analysis

The single genomic 16S rRNA gene sequence of *L. illini* 3055^T^ was compared using NCBI BLAST [18,19] under default settings (e.g., considering only the high-scoring segment pairs (HSPs) from the best 250 hits) with the most recent release of the Greengenes database [20] and the relative frequencies of taxa and keywords (reduced to their stem [21]) were determined, weighted by BLAST scores. The most frequently occurring genera were *Leptospira* (53.4%), *Anaeromyxobacter* (31.6%), *Leptonema* (11.5%), *Turneriella* (1.3%) and *Desulfomonile* (0.8%) (96 hits in total). Regarding the three hits to sequences from members of the species, the average identity within HSPs was 99.7%, whereas the average coverage by HSPs was 97.4%. Among all other species, the one yielding the highest score was *Leptospira wolbachii* (AY631890), which corresponded to an identity of 86.4% and an HSP coverage of 76.8%. (Note that the Greengenes database uses the INSDC (= EMBL/NCBI/DDBJ) annotation, which is not an authoritative source for nomenclature or classification.) The highest-scoring environmental sequence was EF648066 (Greengenes short name 'dynamics during produced water treatment aerobic activated sludge clone HB63'), which showed an identity of 99.2% and an HSP coverage of 98.4%. The most frequently occurring keywords within the labels of all environmental samples which yielded hits were 'microbi' (5.2%), 'soil' (2.3%), 'anaerob' (2.3%), 'industri' (2.0%) and 'ecolog' (1.4%) (154 hits in total). The most frequently occurring keywords within the labels of those environmental samples which yielded hits of a higher score than the highest scoring species were 'microbi' (4.5%), 'cell' (3.1%), 'prmr' (3.0%), 'sediment' (3.0%) and 'coral' (3.0%) (12 hits in total). None of these keywords provides useful information about the close relatives of strain 3055^T^ in the environment.

**Figure 1:**
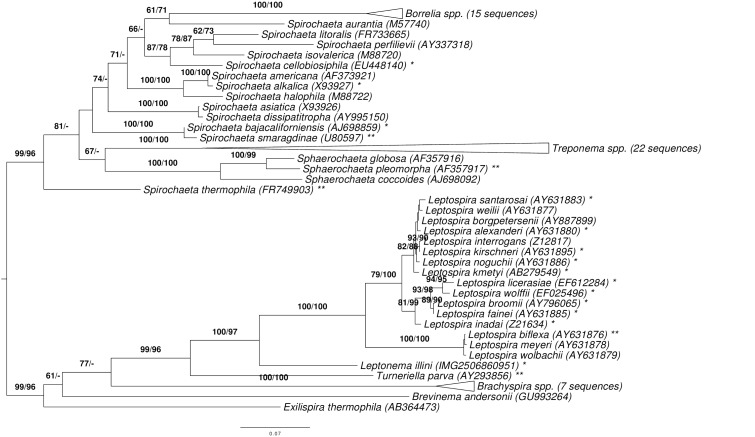
Phylogenetic tree highlighting the position of *L. illini* relative to the type strains of the other species within the phylum *Spirochaetes*. The tree was inferred from 1,325 aligned characters [22,23] of the 16S rRNA gene sequence under the maximum likelihood (ML) criterion [24]. Rooting was done initially using the midpoint method [25] and then checked for its agreement with the current classification (Table 1). The branches are scaled in terms of the expected number of substitutions per site. Numbers adjacent to the branches are support values from 550 ML bootstrap replicates [26] (left) and from 1,000 maximum-parsimony bootstrap replicates [27] (right) if larger than 60%. Lineages with type strain genome sequencing projects registered in GOLD [28] are labeled with one asterisk. Those also listed as 'Complete and Published' with two asterisks (see [29-35] and CP003155 for *Sphaerochaeta pleomorpha*, CP002903 for *Spirochaeta thermophila*, CP002696 for *Treponema brennaborense,* CP001841 for *T. azotonutricium*). The collapsed *Treponema* subtree contains three species formerly assigned to *Spirochaeta* that have recently been included in the genus *Treponema*, even though those names are not yet validly published [34].

Figure 1 shows the phylogenetic neighborhood of *L. illini* in a 16S rRNA based tree. The sequence of the single 16S rRNA gene copy in the genome does not differ from the previously published 16S rRNA sequence (AY714984).

**Table 1:** Classification and general features of *L. illini* 3055^T^ according to the MIGS recommendations [36].

**MIGS ID**	**Property**	**Term**	**Evidence code**
		Domain *Bacteria*	TAS [37]
		Phylum *Spirochaetes*	TAS [38]
		Class *Spirochaetes*	TAS [39,40]
	Current classification	Order *Spirochaetales*	TAS [41,42]
		Family *Leptospiraceae*	TAS [4,14,42]
		Genus *Leptonema*	TAS [4,7]
		Species *Leptonema illini*	TAS [4,7]
MIGS-7	Subspecific genetic lineage (strain)	3055^T^	TAS [4]
MIGS-12	Reference for biomaterial	Hovind-Hougen, 1979	TAS [4]
	Gram stain	negative	TAS [4]
	Cell shape	helical rods	TAS [4]
	Motility	motile	TAS [4]
	Sporulation	non-sporulating	TAS [4
	Temperature range	mesophile	TAS [4]
	Optimum temperature	29° C	TAS [4]
	Salinity	not reported	
MIGS-22	Relationship to oxygen	aerobe	TAS [4]
	Carbon source	long-chain fatty acids	TAS [4]
	Energy metabolism	chemoorganotroph	TAS [4]
MIGS-6	Habitat	not specified	
MIGS-6.2	pH	not reported	
MIGS-15	Biotic relationship	free living	TAS [4]
MIGS-14	Known pathogenicity	opportunistic infections	TAS [43]
MIGS-16	Specific host	*Bos taurus* (cow)	TAS [4]
MIGS-18	Health status of host	healthy	TAS [4]
	Biosafety level	1	TAS [44]
MIGS-19	Trophic level	not reported	
MIGS-23.1	Isolation	urine of a bull	TAS [4]
MIGS-4	Geographic location	Iowa	TAS [5]
MIGS-5	Time of sample collection	1965	TAS [1]
MIGS-4.1	Latitude	not reported	
MIGS-4.2	Longitude	not reported	
MIGS-4.3	Depth	not reported	
MIGS-4.4	Altitude	not reported	

Evidence codes - TAS: Traceable Author Statement (i.e., a direct report exists in the literature); NAS: Non-traceable Author Statement (i.e., not directly observed for the living, isolated sample, but based on a generally accepted property for the species, or anecdotal evidence). Evidence codes are from the Gene Ontology project [45].

### Morphology and physiology

**Figure 2:**
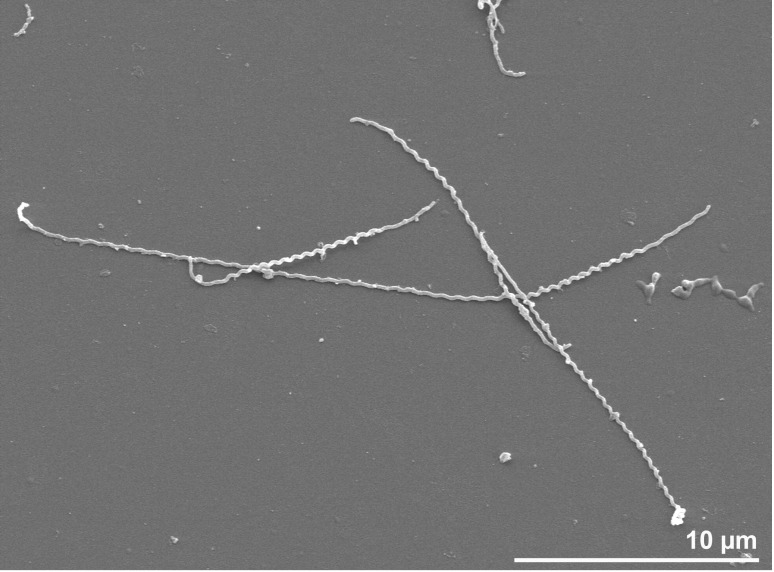
Scanning electron micrograph of *L. illini* 3055^T^

The unicellular cells of strain 3055^T^ stain Gram negatively and are of helical shape (13-21 µm long and 0.1 µm wide) [4] [Figure 2]. Most cells have hook-shaped ends and display a typical leptospiral morphology [46]. The wavelength of the coils within the helix is about 0.6 µm with an amplitude of about 0.1 µm. A single flagellum is inserted at each pole and in well-preserved cells the flagellum is entwined with the helical body within the periplasmatic cell for about four to six turns of the helix (not visible in Figure 2). Rotation of the flagella by a flagellar motor induces changes in the cell morphology that drives motility [47]. In cells treated with Myxobacter Al-1 protease [48] bundles of three to four cytoplasmic tubules are observed which originate close to the insertion point of each of the two flagella. The bundles are located close to the inner site of the cytoplasmic membrane just underneath the flagellum. As bundles and flagella are shorter than the total length of the cell, the middle part is devoid of both. Flagella, released by the AL-1 protease, are often found as spirals. Each flagellum consists of a core (diameter 10 nm), covered by a sheath (diameter 16 nm). One of the arguments to classify strain 3055 as the type of a new genus was the structure of the insertion part of the flagellum, similar to those of Gram-positive bacteria in *L. illini* while other leptospiras possess the Gram-negative type insertion [4].

Serum and long-chain fatty acids are required for growth, no serum is required in trypticase soy broth. The organism is chemoorganotrophic and aerobic. Long-chain fatty acids (>14 carbons) are used as source of carbon and energy. Ammonia, in the form of inorganic salts rather than amino acids is used as a nitrogen source. Purines, but not pyrimidines, are utilized. Strain 3005^T^ is non-pathogenic for hamsters, mice, gerbils, guinea pigs and cattle [15], although it may cause opportunistic infections, as it has been isolated from the blood of a HIV-infected patient [43].

### Chemotaxonomy

No data are available for fatty acids, quinones or polar lipids. The G+C content of the DNA was previously reported with 51-53 mol% [49], which is below the value inferred from the genome sequence (see genome statistics table).

## Genome sequencing and annotation

### Genome project history

This organism was selected for sequencing on the basis of its phylogenetic position [50], and is part of the ***G****enomic* ***E****ncyclopedia of* ***Bacteria**** and* ***Archaea***** project [51]. The genome project is deposited in the Genomes OnLine Database [28] and the complete genome sequence is deposited in GenBank. Sequencing, finishing and annotation were performed by the DOE Joint Genome Institute (JGI) using state of the art sequencing technology [52]. A summary of the project information is shown in Table 2.

**Table 2:** Genome sequencing project information

MIGS ID	Property	Term
MIGS-31	Finishing quality	Improved high quality draft
MIGS-28	Libraries used	Three genomic libraries: one 454 pyrosequence standard library, two 454 PE library (13 kb insert size), one Illumina library
MIGS-29	Sequencing platforms	Illumina GAii, 454 GS FLX Titanium
MIGS-31.2	Sequencing coverage	1,276.9 × Illumina; 35.5 × pyrosequence
MIGS-30	Assemblers	Newbler version 2.3, Velvet 1.0.13, phrap version SPS - 4.24
MIGS-32	Gene calling method	Prodigal 1.4, GenePRIMP
	INSDC ID	AHKT00000000
	GenBank Date of Release	January 24, 2012
	GOLD ID	Gi04604
	NCBI project ID	60435
	Database: IMG	2506783010
MIGS-13	Source material identifier	DSM 21528
	Project relevance	Tree of Life, GEBA

### Growth conditions and DNA isolation

*L. illini* strain 3055^T^, DSM 21528, was grown in DSMZ medium 1113 (*Leptospira* Medium) at 30°C. DNA was isolated from 1-1.5 g of cell paste using MasterPure Gram-positive DNA purification kit (Epicentre MGP04100) following the standard protocol as recommended by the manufacturer with modification st/DL for cell lysis as described in Wu *et al*. 2009 [51]. DNA is available through the DNA Bank Network [53].

### Genome sequencing and assembly

The genome was sequenced using a combination of Illumina and 454 sequencing platforms. All general aspects of library construction and sequencing can be found at the JGI website [54]. Pyrosequencing reads were assembled using the Newbler assembler (Roche). The initial Newbler assembly consisting of 140 contigs in tree scaffolds was converted into a phrap [55] assembly by making fake reads from the consensus, to collect the read pairs in the 454 paired end library. Illumina GAii sequencing data (5,940 Mb) was assembled with Velvet [56] and the consensus sequences were shredded into 1.5 kb overlapped fake reads and assembled together with the 454 data. The 454 draft assembly was based on 179 Mb 454 draft data and all of the 454 paired end data. Newbler parameters are -consed -a 50 -l 350 -g -m -ml 20. The Phred/Phrap/Consed software package [55] was used for sequence assembly and quality assessment in the subsequent finishing process. After the shotgun stage, reads were assembled with parallel phrap (High Performance Software, LLC). Possible mis-assemblies were corrected with gapResolution [54], Dupfinisher [57], or sequencing cloned bridging PCR fragments with subcloning. Gaps between contigs were closed by editing in Consed, by PCR and by Bubble PCR primer walks (J.-F. Chang, unpublished). A total of 103 additional reactions and one shatter library were necessary to close gaps and to raise the quality of the finished sequence. Illumina reads were also used to correct potential base errors and increase consensus quality using a software Polisher developed at JGI [58]. The error rate of the completed genome sequence is less than 1 in 100,000. Together, the combination of the Illumina and 454 sequencing platforms provided 1,312.4 × coverage of the genome. The final assembly contained 488,975 pyrosequence and 75,603,747 Illumina reads.

### Genome annotation

Genes were identified using Prodigal [59] as part of the DOE-JGI genome annotation pipeline [60], followed by a round of manual curation using the JGI GenePRIMP pipeline [61]. The predicted CDSs were translated and used to search the National Center for Biotechnology Information (NCBI) non-redundant database, UniProt, TIGR-Fam, Pfam, PRIAM, KEGG, COG, and InterPro databases. Additional gene prediction analysis and functional annotation was performed within the Integrated Microbial Genomes - Expert Review (IMG-ER) platform [62].

## Genome properties

**Figure 3:**
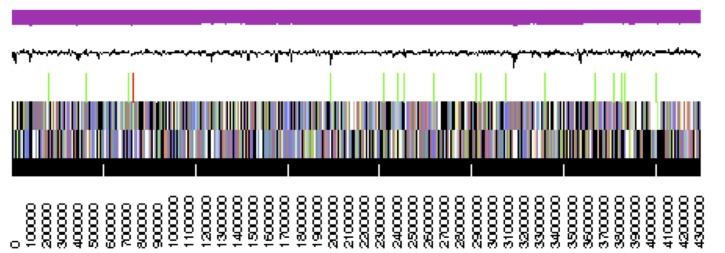
Graphical map of the largest scaffold. From bottom to the top: Genes on forward strand (color by COG categories), Genes on reverse strand (color by COG categories), RNA genes (tRNAs green, rRNAs red, other RNAs black), GC content, GC skew (purple/olive).

The genome statistics are provided in Table 3 and Figure 3. The assembly of the draft genome sequence consists of three scaffolds with 4,325,094 bp, 184,087 bp and 13,579 bp length, respectively, and a G+C content of 54.3%. Of the 4,277 genes predicted, 4,230 were protein-coding genes, and 47 RNAs; 69 pseudogenes were also identified. The majority of the protein-coding genes (60.3%) were assigned a putative function while the remaining ones were annotated as hypothetical proteins. The distribution of genes into COGs functional categories is presented in Table 4.

**Table 3:** Genome Statistics

**Attribute**	**Value**	**% of Total**
Genome size (bp)	4,522,760	100.00
DNA coding region (bp)	4,079,818	90.21
DNA G+C content (bp)	2,453,341	54.26
Number of scaffolds	3	
Extrachromosomal elements	unknown	
Total genes	4,277	100.00
RNA genes	47	1.10
rRNA operons	1	
tRNA genes	41	0.96
Protein-coding genes	4,230	98.90
Pseudo genes	69	1.61
Genes with function prediction	2,579	60.30
Genes in paralog clusters	1,764	41.24
Genes assigned to COGs	2,805	65.58
Genes assigned Pfam domains	2,865	66.99
Genes with signal peptides	1,481	34.63
Genes with transmembrane helices	1,089	25.46
CRISPR repeats	0	

**Table 4:** Number of genes associated with the general COG functional categories

**Code**	**Value**	**%age**	**Description**
J	156	5.0	Translation, ribosomal structure and biogenesis
A	0	0.0	RNA processing and modification
K	201	6.5	Transcription
L	194	6.3	Replication, recombination and repair
B	4	0.1	Chromatin structure and dynamics
D	34	1.1	Cell cycle control, cell division, chromosome partitioning
Y	0	0.0	Nuclear structure
V	61	2.0	Defense mechanisms
T	303	9.8	Signal transduction mechanisms
M	226	7.3	Cell wall/membrane/envelope biogenesis
N	108	3.5	Cell motility
Z	0	0.0	Cytoskeleton
W	0	0.0	Extracellular structures
U	74	2.4	Intracellular trafficking, secretion, and vesicular transport
O	119	3.8	Posttranslational modification, protein turnover, chaperones
C	160	5.2	Energy production and conversion
G	111	3.6	Carbohydrate transport and metabolism
E	189	6.1	Amino acid transport and metabolism
F	60	1.9	Nucleotide transport and metabolism
H	139	4.5	Coenzyme transport and metabolism
I	131	4.2	Lipid transport and metabolism
P	128	4.1	Inorganic ion transport and metabolism
Q	43	1.4	Secondary metabolites biosynthesis, transport and catabolism
R	401	12.9	General function prediction only
S	260	8.4	Function unknown
-	1,472	34.4	Not in COGs

## References

[1] Tripathy DN Hanson LE Colonial and morphologic variations of *Leptospira illini* strain 3055. Am J Vet Res 1972; 33:1723-1727 5047125

[2] Tripathy DN Hanson LE Studies of *Leptospira illini,* strain 3055: pathogenicity for different animals. Am J Vet Res 1973; 34:557-562 4693813

[3] Tripathy DN Hanson LE Studies of *Leptospira illini,* strain 3055: immunological and serological determinations. Am J Vet Res 1973; 34:563-565 4632748

[4] Hovind-Hougen K *Leptospiraceae*, a new family to include *Leptospira* Noguchi 1917 and *Leptonema* gen. nov. Int J Syst Bacteriol 1979; 29:245-251 10.1099/00207713-29-3-245

[5] Hanson LE Tripathy DN Evans LB Alexander AD An unusual *Leptospira*, serotype *illini* (a new serotype). Int J Syst Bacteriol 1974; 24:355-357 10.1099/00207713-24-3-355

[6] Skerman VBD McGowan V Sneath PHA Approved Lists of Bacterial Names. Int J Syst Bacteriol 1980; 30:225-420 10.1099/00207713-30-1-225 20806452

[7] Validation of the Publication of New Names and New Combinations Previously Effectively Published Outside the IJSB List No 10. Int J Syst Bacteriol 1983; 33:438-440 10.1099/00207713-33-2-438 9336942

[8] Paster BJ Stackebrandt E Hespell RB Hahn CM Woese CR The phylogeny of the spirochetes. Syst Appl Microbiol 1984; 5:337-351 10.1016/S0723-2020(84)80036-3 11541974

[9] Paster BJ, Dewhirst FE, Weisburg WG, Tordoff LA, Fraser GJ, Hespell RB, Stanton TB, Zablen L, Mandelco L, Woese CR. Phylogenetic analysis of the spirochetes. J Bacteriol 1991; 173:6101-6109 1917844 10.1128/jb.173.19.6101-6109.1991 PMC208357

[10] Paster BJ Dewhirst FE Phylogenetic foundation of spirochetes. J Mol Microbiol Biotechnol 2000; 2:341-244 11075904

[11] Ramadass P Jarvis BDW Corner RJ Cinco M Marshall RB DNA relatedness among strains of *Leptospira biflexa.* Int J Syst Bacteriol 1990; 40:231-235 10.1099/00207713-40-3-231 2397191

[12] Morey RE Galloway RL Bragg SL Steigerwalt AG Mayer LW Levett PN Species-specific identification of *Leptospiraceae* by 16S rRNA gene sequencing. J Clin Microbiol 2006; 44:3510-3516 10.1128/JCM.00670-06 17021075 PMC1594759

[13] Ramadass P Jarvis BDW Corner RJ Penny D Marshall RB Genetic characterization of pathogenic *Leptospira* species by DNA hybridization. Int J Syst Bacteriol 1992; 42:215-219 10.1099/00207713-42-2-215 1581182

[14] Levett PN Morey RE Galloway R Steigerwalt AG Ellis WA Reclassification of *Leptospira parva* Hovind-Hougen *et al* 1982 as *Turneriella parva* gen. nov., comb. nov. Int J Syst Evol Microbiol 2005; 55:1497-1499 10.1099/ijs.0.63088-0 16014471

[15] Hookey JV Characterization of *Leptospiraceae* by 16S DNA restriction fragment length polymorphisms. J Gen Microbiol 1993; 139:1681-1689 10.1099/00221287-139-8-1681 7691982

[16] Woo TH Smythe LD Symonds ML Norris MA Dohnt MF Patel BK Rapid distinction between *Leptonema* and *Leptospira* by PCR amplification of 16S-23S ribosomal DNA spacer. FEMS Microbiol Lett 1996; 142:85-90 10.1111/j.1574-6968.1996.tb08412.x 8759793

[17] Woo TH Patel BKC Cinco M Smythe LD Symonds ML Norris MA Dohnt MF Real-time homogeneous assay of rapid cycle polymerase chain reaction product for identification of *Leptonema illini.* Anal Biochem 1998; 259:112-117 10.1006/abio.1997.2532 9606151

[18] Altschul SF Gish W Miller W Myers EW Lipman DJ Basic local alignment search tool. J Mol Biol 1990; 215:403-410 2231712 10.1016/S0022-2836(05)80360-2

[19] Korf I, Yandell M, Bedell J. BLAST, O'Reilly, Sebastopol, 2003.

[20] DeSantis TZ Hugenholtz P Larsen N Rojas M Brodie EL Keller K Huber T Dalevi D Hu P Andersen GL Greengenes, a chimera-checked 16S rRNA gene database and workbench compatible with ARB. Appl Environ Microbiol 2006; 72:5069-5072 10.1128/AEM.03006-05 16820507 PMC1489311

[21] Porter MF. An algorithm for suffix stripping. *Program: electronic library and information systems* 1980; **14**:130-137.

[22] Lee C Grasso C Sharlow MF Multiple sequence alignment using partial order graphs. Bioinformatics 2002; 18:452-464 10.1093/bioinformatics/18.3.452 11934745

[23] Castresana J Selection of conserved blocks from multiple alignments for their use in phylogenetic analysis. Mol Biol Evol 2000; 17:540-552 10.1093/oxfordjournals.molbev.a026334 10742046

[24] Stamatakis A Hoover P Rougemont J A rapid bootstrap algorithm for the RAxML web-servers. Syst Biol 2008; 57:758-771 10.1080/10635150802429642 18853362

[25] Hess PN De Moraes Russo CA An empirical test of the midpoint rooting method. Biol J Linn Soc Lond 2007; 92:669-674 10.1111/j.1095-8312.2007.00864.x PMC7110036 32287391

[26] Pattengale ND Alipour M Bininda-Emonds ORP Moret BME Stamatakis A How many bootstrap replicates are necessary? Lect Notes Comput Sci 2009; 5541:184-200 10.1007/978-3-642-02008-7_13

[27] Swofford DL. PAUP*: Phylogenetic Analysis Using Parsimony (*and Other Methods), Version 4.0 b10. Sinauer Associates, Sunderland, 2002.

[28] Pagani I Liolios K Jansson J Chen IM Smirnova T Nosrat B Markowitz VM Kyrpides NC The Genomes OnLine Database (GOLD) v.4: status of genomic and metagenomic projects and their associated metadata. Nucleic Acids Res 2012; 40:D571-D579 10.1093/nar/gkr1100 22135293 PMC3245063

[29] Abt B Han C Scheuner C Lu M Lapidus A Nolan M Lucas S Hammon N Deshpande S Cheng JF Complete genome sequence of the termite hindgut bacterium *Spirochaeta coccoides* type strain (SPN1^T^), reclassification in the genus *Sphaerochaeta* as *Sphaerochaeta coccoides* comb. nov. and emendations of the family *Spirochaetaceae* and the genus *Sphaerochaeta.* Stand Genomic Sci 2012; 6:194-209 10.4056/sigs.2796069 22768363 PMC3388779

[30] Han C Gronow S Teshima H Lapidus A Nolan M Lucas S Hammon N Deshpande S Cheng JF Zeytun A Complete genome sequence of *Treponema succinifaciens* type strain (6091^T^). Stand Genomic Sci 2011; 4:361-370 10.4056/sigs.1984594 21886863 PMC3156407

[31] Mavromatis K Yasawong M Chertkov O Lapidus A Lucas S Nolan M Glavina del Rio T Tice H Cheng JF Pitluck S Complete genome sequence of *Spirochaeta smaragdinae* type strain (SEBR 4228^T^). Stand Genomic Sci 2010; 3:136-144 21304743 10.4056/sigs.1143106 PMC3035371

[32] Pati A Sikorski J Gronow S Lapidus A Copeland A Glavina del Rio T Nolan M Lucas S Chen F Tice H Complete genome sequence of *Brachyspira murdochii* type strain (56-150^T^). Stand Genomic Sci 2010; 2:260-269 10.4056/sigs.831993 21304710 PMC3035287

[33] Fraser CM Casjens S Huang WM Sutton GG Clayton RA Lathigra R White O Ketchum KA Dodson R Hickey EK Genomic sequence of a Lyme disease spirochaete, *Borrelia burgdorferi.* Nature 1997; 390:580-586 10.1038/37551 9403685

[34] Abt B Göker MG Scheuner C Han C Lu M Misra M Lapidus A Nolan M Lucas S Hammon N Genome sequence of the thermophilic fresh-water bacterium *Spirochaeta caldaria* type strain (H1^T^), reclassification of *Spirochaeta caldaria* and *Spirochaeta stenostrepta* in the genus *Treponema* as *Treponema caldaria* comb. nov. and *Treponema stenostrepta* comb. nov., revival of the name *Treponema zuelzerae* comb. nov., and emendation of the genus *Treponema.* Stand Genomic Sci 2013; 8:88-105 10.4056/sigs.3096473 23961314 PMC3739177

[35] Stackebrandt E Chertkov O Lapidus A Nolan M Lucas S Hammon N Deshpande S Cheng JF Tapia R Goodwin LA Genome sequence of the free-living aerobe spirochaete *Turneriella parva* type strain (H^T^), end emendation of *Turneriella parva.* Stand Genomic Sci 2013; (this issue). 10.4056/sigs.3617113 PMC3746428 23991255

[36] Field D Garrity G Gray T Morrison N Selengut J Sterk P Tatusova T Thomson N Allen MJ Angiuoli SV The minimum information about a genome sequence (MIGS) specification. Nat Biotechnol 2008; 26:541-547 10.1038/nbt1360 18464787 PMC2409278

[37] Woese CR Kandler O Wheelis ML Towards a natural system of organisms: proposal for the domains *Archaea, Bacteria*, and *Eucarya.* Proc Natl Acad Sci USA 1990; 87:4576-4579 10.1073/pnas.87.12.4576 2112744 PMC54159

[38] Garrity GM, Holt JG. The Road Map to the Manual. *In:* Garrity GM, Boone DR, Castenholz RW (*eds*), Bergey's Manual of Systematic Bacteriology, Second Edition, Volume 1, Springer, New York, 2001, p. 119-169.

[39] Ludwig W, Euzeby J, Whitman WG. Draft taxonomic outline of the *Bacteroidetes, Planctomycetes, Chlamydiae, Spirochaetes, Fibrobacteres, Fusobacteria, Acidobacteria, Verrucomicrobia, Dictyoglomi*, and *Gemmatimonadetes* http://www.bergeys.org/outlines/Bergeys_Vol_4_Outline.pdf Taxonomic Outline 2008.

[40] Judicial Commission of the International Committee on Systematics of Prokaryotes The nomenclatural types of the orders *Acholeplasmatales, Halanaerobiales*, Halobacteriales, *Methanobacteriales, Methanococcales, Methanomicrobiales, Planctomycetales, Prochlorales, Sulfolobales, Thermococcales, Thermoproteales* and *Verrucomicrobiales* are the genera *Acholeplasma, Halanaerobium, Halobacterium, Methanobacterium, Methanococcus, Methanomicrobium, Planctomyces, Prochloron, Sulfolobus, Thermococcus, Thermoproteus* and *Verrucomicrobium*, respectively. Opinion 79. Int J Syst Evol Microbiol 2005; 55:517-518 10.1099/ijs.0.63548-0 15653928

[41] Buchanan RE Studies in the nomenclature and classification of bacteria. II. The primary subdivisions of the *Schizomycetes.* J Bacteriol 1917; 2:155-164 16558735 10.1128/jb.2.2.155-164.1917 PMC378699

[42] Skerman VBD McGowan V Sneath PHA Approved Lists of Bacterial Names. Int J Syst Bacteriol 1980; 30:225-420 10.1099/00207713-30-1-225 20806452

[43] Rocha T Cardoso EA Terrinha AM Nunes JFM Hovind-Hougen K Cinco M Isolation of a new serovar of the genus *Leptonema* in the family *Leptospiraceae. Zbl.* Bakt. 1993; 279:167-172 10.1016/s0934-8840(11)80394-4 8219489

[44] BAuA 2010 – 2012 update, Classification of bacteria and archaea in risk groups. http://www.baua.de TRBA 466, p. 19.

[45] Ashburner M Ball CA Blake JA Botstein D Butler H Cherry JM Davis AP Dolinski K Dwight SS Eppig JT Gene ontology: tool for the unification of biology. Nat Genet 2000; 25:25-29 10.1038/75556 10802651 PMC3037419

[46] Hovind-Hougen K Determination by means of electron microscopy of morphological criteria of value for classification of some spirochetes, in particular treponemes. Acta Pathol Microbiol Scand. Sect. B. Suppl. 1976; 255:28-30 58539

[47] Wolgemuth CW Charon NW Goldstein SF Goldstein RE The flagellar cytoskeleton of the spirochetes. J Mol Microbiol Biotechnol 2006; 11:221-227 10.1159/000094056 16983197

[48] Hovind-Hougen K Birch-Andersen A Electron microscopy of endoflagella and microtubules in *Treponema* Reiter. Acta Pathol Microbiol Scand Sect B 1971; 79:37-50 4102475 10.1111/j.1699-0463.1971.tb00031.x

[49] Brendle JJ Rogul M Alexander AD Deoxyribonucleic acid hybridization among selected leptospiral serotypes. Int J Syst Bacteriol 1974; 24:205-214 10.1099/00207713-24-2-205

[50] Klenk HP Göker M *En route* to a genome-based classification of *Archaea* and *Bacteria*? Syst Appl Microbiol 2010; 33:175-182 10.1016/j.syapm.2010.03.003 20409658

[51] Wu D Hugenholtz P Mavromatis K Pukall R Dalin E Ivanova NN Kunin V Goodwin L Wu M Tindall BJ A phylogeny-driven genomic encyclopaedia of *Bacteria* and *Archaea.* Nature 2009; 462:1056-1060 10.1038/nature08656 20033048 PMC3073058

[52] Mavromatis K Land ML Brettin TS Quest DJ Copeland A Clum A Goodwin L Woyke T Lapidus A Klenk HP The fast changing landscape of sequencing technologies and their impact on microbial genome assemblies and annotation. PLoS ONE 2012; 7:e48837 10.1371/journal.pone.0048837 23251337 PMC3520994

[53] Gemeinholzer B Dröge G Zetzsche H Haszprunar G Klenk HP Güntsch A Berendsohn WG Wägele JW The DNA Bank Network: the start from a German initiative. Biopreserv Biobank 2011; 9:51-55 10.1089/bio.2010.0029 24850206

[54] JGI website. http://www.jgi.doe.gov/

[55] The Phred/Phrap/Consed software package. http://www.phrap.com

[56] Zerbino DR Birney E Velvet: algorithms for de novo short read assembly using de Bruijn graphs. Genome Res 2008; 18:821-829 10.1101/gr.074492.107 18349386 PMC2336801

[57] Han C, Chain P. Finishing repeat regions automatically with Dupfinisher. *In:* Proceeding of the 2006 international conference on bioinformatics & computational biology. Arabnia HR, Valafar H (*eds*), CSREA Press. June 26-29, 2006: 141-146.

[58] Lapidus A, LaButti K, Foster B, Lowry S, Trong S, Goltsman E. POLISHER: An effective tool for using ultra short reads in microbial genome assembly and finishing. AGBT, Marco Island, FL, 2008.

[59] Hyatt D Chen GL LoCascio PF Land ML Larimer FW Hauser LJ Prodigal: prokaryotic gene recognition and translation initiation site identification. BMC Bioinformatics 2010; 11:119 10.1186/1471-2105-11-119 20211023 PMC2848648

[60] Mavromatis K Ivanova NN Chen IM Szeto E Markowitz VM Kyrpides NC The DOE-JGI Standard operating procedure for the annotations of microbial genomes. Stand Genomic Sci 2009; 1:63-67 10.4056/sigs.632 21304638 PMC3035208

[61] Pati A Ivanova NN Mikhailova N Ovchinnikova G Hooper SD Lykidis A Kyrpides NC GenePRIMP: a gene prediction improvement pipeline for prokaryotic genomes. Nat Methods 2010; 7:455-457 10.1038/nmeth.1457 20436475

[62] Markowitz VM Ivanova NN Chen IMA Chu K Kyrpides NC IMG ER: a system for microbial genome annotation expert review and curation. Bioinformatics 2009; 25:2271-2278 10.1093/bioinformatics/btp393 19561336

